# Extract of a Report on the Means of Purifying the Air in Sick Chambers, from Infection

**Published:** 1799-05

**Authors:** J. B. Van Mons


### *Extract of a Report on the means of purifying the Air in Sick Chambers, from Infection—By* J. B. Van Mons.

(From the Transactions of the Medical Society of Brussels.)

I HAVE in general found the air in the chambers of ſick perſons compoſed of a quantity of carbonic acid gas, hydrogen gas, oxygen and azote, with, ſometimes, a ſmall portion of ammonical gas, and of that particular emanation, which is called contagious miaſma, and which appears to be a peculiar combination of carbonated hydrogen gas, with thoſe particles, the nature of which is as yet little known. The hydrogen gas, however, generally holds in ſolution, pure carbon, phoſphorus, &c. hence ariſes the diſagreeable ſmell of theſe airs. The carbonic acid gas would form a much more conſiderable part of the air which ſurrounds the ſick, if that gas were not continually condenſed, either by the ammonia generated and diſengaged in all diſeaſes, or by the decompoſition of animal ſubſtances containing azote. We have ſeen into what contradictions thoſe have fallen, who have admitted in the air, at the ſame time, the carbonic and ammoniacal gas. We have, in like manner, ſeen the want of juſt concluſion, in expoſing veſſels filled with lime-water, in chambers, the inconvenience of which is, in every caſe, to leave or introduce into the air, the ammoniacal gas, by the abſorption absorption of the carbonic gas, and sometimes to condense an irrespirable gas.

I am satisfied, that in a state of health we form more water, in sickness more carbonic acid. The carbon appears to require a certain temperature, to exercise a stronger attraction upon the oxygen, than the hydrogen.

*(The remaining part of this article is occupied with the means of purifying infected air, and of adding to it a portion of respirable air.)*

Among the first means, I class water in a state of vapour, which is hurtful to few sick persons, and which, being a better solvent, precipitates the volatile and putrid emanations, more readily than the muriatic and acetous acids. When the air is surcharged with ammonia, I think it better to disengage it by means of the carbonic acid, than by vinegar or any other acid. The case, in which Citizen Guyton successively employed the gazeous muriatic acid, is altogether different from those of which we now treat.

The sulphurous acid gas might be, in some cases, useful for decomposing miasmata, by overcoming them with its oxygen; but it leaves behind an oxyd of sulphur, the smell of which is very injurious. The oxygenous muriatic gas is threrefore preferable.

The gases which I consider as increasing the irrespirable portion of air, are the carbonic acid, and the hydrogen. The former ought to be passed hrough water, and the latter through oil. The carbonic acid gas is by this process deprived of the portion of acid which is employed to disengage it, and the carbon is precipitated from the hydrogenous gas, which holds it in solution. The oil, after having been for some time thus used, becomes quite black, and is changed into empyreumatic or carbonated oil.

